# *In-silico* formulation of a next-generation polyvalent vaccine against multiple strains of monkeypox virus and other related poxviruses

**DOI:** 10.1371/journal.pone.0300778

**Published:** 2024-05-17

**Authors:** Abu Tayab Moin, Nurul Amin Rani, Rajesh B. Patil, Tanjin Barketullah Robin, Md. Asad Ullah, Zahidur Rahim, Md. Foyzur Rahman, Talha Zubair, Mohabbat Hossain, A. K. M. Moniruzzaman Mollah, Nurul Absar, Mahboob Hossain, Mohammed Abul Manchur, Nazneen Naher Islam

**Affiliations:** 1 Faculty of Biological Sciences, Department of Genetic Engineering and Biotechnology, Laboratory of Clinical Genetics, Genomics and Enzyme Research (LCGGER), University of Chittagong, Chattogram, Bangladesh; 2 Faculty of Biotechnology and Genetic Engineering, Sylhet Agricultural University, Sylhet, Bangladesh; 3 Department of Pharmaceutical Chemistry, Sinhgad Technical Education Society’s, Sinhgad College of Pharmacy, Maharashtra, India; 4 Faculty of Biological Sciences, Department of Biotechnology and Genetic Engineering, Jahangirnagar University, Savar, Dhaka, Bangladesh; 5 Department of Zoology, Jahangirnagar University, Dhaka, Bangladesh; 6 Department of Pharmacy, Dhaka International University, Dhaka, Bangladesh; 7 Notre Dame College, Dhaka, Bangladesh; 8 Department of Biological Sciences, Asian University for Women (AUW), Chattogram, Bangladesh; 9 Faculty of Basic Medical and Pharmaceutical Sciences, Department of Biochemistry and Biotechnology, University of Science & Technology Chittagong, Khulshi, Chittagong, Bangladesh; 10 Department of Mathematics and Natural Sciences, Microbiology Program, School of Data and Sciences, BRAC University, Dhaka, Bangladesh; 11 Faculty of Biological Sciences, Department of Microbiology, University of Chittagong, Chattogram, Bangladesh; The University of Texas Medical Branch at Galveston, UNITED STATES

## Abstract

Mpox (formerly known as monkeypox) virus and some related poxviruses including smallpox virus pose a significant threat to public health, and effective prevention and treatment strategies are needed. This study utilized a reverse vaccinology approach to retrieve conserved epitopes for monkeypox virus and construct a vaccine that could provide cross-protection against related viruses with similar antigenic properties. The selected virulent proteins of monkeypox virus, MPXVgp165, and Virion core protein P4a, were subjected to epitope mapping for vaccine construction. Two vaccines were constructed using selected T cell epitopes and B cell epitopes with PADRE and human beta-defensins adjuvants conjugated in the vaccine sequence. Both constructs were found to be highly antigenic, non-allergenic, nontoxic, and soluble, suggesting their potential to generate an adequate immune response and be safe for humans. Vaccine construct 1 was selected for molecular dynamic simulation studies. The simulation studies revealed that the TLR8-vaccine complex was more stable than the TLR3-vaccine complex. The lower RMSD and RMSF values of the TLR8 bound vaccine compared to the TLR3 bound vaccine suggested better stability and consistency of hydrogen bonds. The Rg values of the vaccine chain bound to TLR8 indicated overall stability, whereas the vaccine chain bound to TLR3 showed deviations throughout the simulation. These results suggest that the constructed vaccine could be a potential preventive measure against monkeypox and related viruses however, further experimental validation is required to confirm these findings.

## 1. Introduction

The *Poxviridae* family comprises double-stranded DNA viruses, including *Orthopoxvirus* that are responsible for causing a diverse array of diseases in humans and animals [1]. *Orthopoxvirus*, a genus within the family *Poxviridae*, comprises several notable viruses, including smallpox virus and monkeypox virus. These viruses share a high degree of genomic similarity, with around 95% of their genomes being conserved, and have been associated with outbreaks of severe disease in humans [2,3]. While smallpox virus, also known as variola virus, caused millions of deaths worldwide, monkeypox virus, although less deadly than smallpox virus, can still cause severe illness in humans and has been identified as a potential emerging infectious disease. *Orthopoxvirus*es remain a significant public health concern due to their potential for bioterrorism and the emergence of new and dangerous strains [4,5]. The *Orthopoxvirus* family encompasses some other closely related viruses such as vaccinia, horsepox, taterapox, cowpox, alaskapox, camelpox, raccoonpox, orthopox, and volepox etc. that have also been associated with outbreaks of zoonotic diseases [6,7]. At the genetic level, these viruses have a double-stranded DNA genome of approximately 130–300 kilobase pairs in length, encoding for a range of structural and non-structural proteins involved in viral replication, immune evasion, and pathogenesis. These viruses also share a conserved set of core genes, including those involved in DNA replication, transcription, and virion assembly [8,9]. At the proteomic level, the structural proteins of these viruses share significant homology, including the major envelope glycoproteins responsible for virion attachment and entry into host cells [10,11]. Additionally, many of these viruses share a similar set of immunomodulatory proteins, such as interferon inhibitors and complement evasion factors that allow them to evade host immune responses [12]. These similarities between *Orthopoxviruses* indicate the potential for cross-reactivity and zoonotic transmission between different virus species, which can lead to the emergence of novel viral strains with altered pathogenicity and host range [13,14]. This highlights the importance of continued surveillance and monitoring of these viruses in both animal and human populations, as well as the development of effective prevention and control measures. Consequently, it is crucial to persist with research efforts to investigate the molecular aspects of immune responses triggered by viral infections, with the goal of designing potent vaccines to combat these viruses [15,16]. Nowadays, peptide-based multi-epitope vaccines are considered more promising than other vaccination strategies for certain diseases. They have multiple advantages, including enhanced safety, efficacy, and specificity. They use short amino acid sequences or epitopes to trigger immune responses against pathogens and can reduce the risk of pathogen escape mutants [17,18]. The use of bioinformatics tools and techniques facilitates in identifying potential epitopes and predicting their immunogenicity, thereby aiding in the development of more effective and specific vaccines with a reduced risk of adverse reactions [19,20]. Hence, in this current study, we analyzed the molecular basis of the immunogenic response induced by viruses utilizing high-throughput bioinformatics approaches. We identified potential epitopes and proposed a vaccine candidate through investigating the molecular mechanisms underlying viral infection and the subsequent immune responses. These results will help to enhance the understanding of vaccine development and can be further validated through experimental approaches.

## 2. Methods

**Fig 1:**
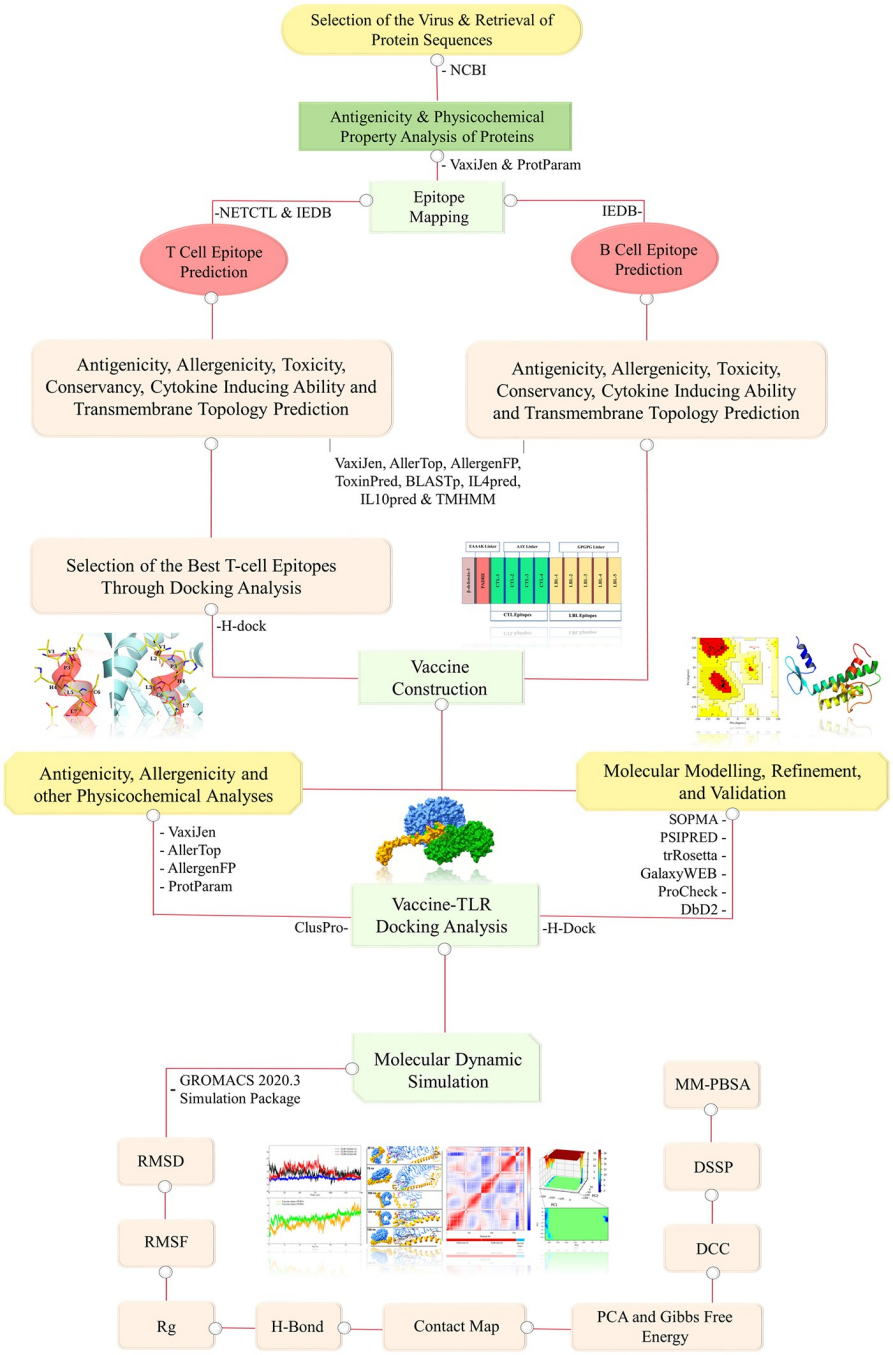
Flowchart of stepwise bioinformatics approaches utilized for vaccine designing.

The stepwise methodology of the entire study is depicted in Fig 1 as a flowchart.

### 2.1. Identification and biophysical property analyses of the proteins

Similar proteins between Monkeypox virus and SPV (smallpox virus) was identified by using the NCBI’s BLASTp tool (https://blast.ncbi.nlm.nih.gov/Blast.cgi) [21]. Where proteins of Monkeypox virus were screened against the smallpox proteins. The similar proteins were retrieved from the NCBI database in FASTA format (http://www.ncbi.nlm.nih.gov/). The selected protein sequence was submitted to the online antigenicity tool VaxiJen v2.0 (http://vaxijen/VaxiJen/VaxiJen.html/) to analyze the antigenic property [22]. Transmembrane topology was assessed by utilizing TMHMM-2.0 server (https://services.healthtech.dtu.dk/service.php?TMHMM-2.0) [23]. Numerous physicochemical properties of the protein were analyzed by utilizing the ExPASy ProtParam server (https://web.expasy.org/protparam//) [24]. Finally, the homologous sequence sets of the chosen antigenic proteins were downloaded from the NCBI database using the BLASTp tool to uncover the conservancy pattern.

### 2.2. Epitope mapping for peptide fusion

The NetCTL prediction method of IEDB (https://www.iedb.org/) was used to identify T cell epitopes. The threshold was chosen at 0.50 allowing the sensitivity at 0.89 and specificity at 0.94. The peptide lengths were set to 9 amino acids for the binding analysis, and all of the MHC class I alleles that were accessible were chosen [25,26]. The PEP-FOLD Peptide Structure Prediction server was used to construct 3D structures of the identified T cell epitopes. The best models were chosen for the docking study. Depending on the accessible structures that have been deposited in the Protein Data Bank (PDB) database, HLA-A*11:01 and HLA-DRB1*04:01 were selected for docking analysis with T cell epitopes respectively [27]. The molecular docking analysis was performed by the H-dock server (http://hdock.phys.hust.edu.cn/) to demonstrate that the proposed epitopes may interact with at least one MHC molecule at minimum binding energy [28]. Once the epitopes were predicted, several analyses were conducted to retrieve the most promising epitopes for vaccine construction. VaxiJen v2.0 server was again used for the determination of epitope antigenicity, TMHMM server was used for the prediction of transmembrane topology, AllergenFP (https://ddg-pharmfac.net/AllergenFP/) and AllerTOP (http://www.ddg-pharmfac.net/AllerTop/) were used for allergenicity prediction [29,30], and finally ToxinPred server (http://crdd.osdd.net/raghava/toxinpred/) for toxicity analysis [31].

### 2.3. Analyses of the biophysical and structural properties of the vaccine

The epitopes with the highest potential for vaccine development are those exhibiting superior antigenicity, non-toxicity, non-allergenicity, and conservancy. To create a final vaccine construct, these epitopes were conjugated using selective linkers (such as EAAAK, AAY, and GPGPG), as well as adjuvants (i.e., PADRE and human beta-defensin (hBds). EAAAK protects the vaccine from degradation, and the addition of linkers stabilised the vaccine design while boosting antigenicity. The PADRE sequence was used as a powerful immunostimulant for improving vaccine potency. By attracting immature dendritic cells, naive memory T cells, and monocytes to the site of infection, the hBD adjuvant strengthens innate and adaptive host defence [32–34]. Antigenicity and allergenicity analyses were conducted again using the same servers as before to confirm the safety and efficacy of the vaccine. The solubility of the vaccine construct upon expression in *Escherichia coli* was assessed using the Protein-Sol server (https://protein-sol.manchester.ac.uk/) [33]. The biophysical properties of the vaccine constructs, including isoelectric pH, aliphatic and instability index, GRAVY values, hydropathicity, estimated half-life, and other properties, were evaluated using the ProtParam tool of the ExPASy server. The secondary structure of the final multi-epitope vaccine was predicted using two servers, SOPMA (https://npsa-prabi.ibcp.fr/cgi-bin/npsa_automat.pl?page=/NPSA/npsa_sopma.html) and PSIPRED (http://bioinf.cs.ucl.ac.uk/psipred/) [35,36]. The tertiary structure of the vaccine constructs was modeled using the trRosetta server (https://yanglab.nankai.edu.cn/trRosetta/), which was then refined by the GalaxyRefine module of the GalaxyWEB server (http://galaxy.seoklab.org/) [37,38]. Finally, the refined models were validated by generating Ramachandran plots, ERRAT score plots, and Z score plots using the SAVES PROCHECK server (https://saves.mbi.ucla.edu/) and ProSA-web server (https://prosa.services.came.sbg.ac.at/prosa.php), respectively. The discontinuous B-cell epitopes of validated 3D structure of the vaccine were predicted using the server ElliPro (http://tools.iedb.org/ellipro/).

### 2.4. Molecular docking and dynamics simulation studies

Molecular docking analysis was performed in order to predict the binding affinity and interaction patterns between the vaccine construct and Tool-like receptors TLR-3 (PDB ID: 7C76) and TLR-8 (PDB ID: 7R52) [39,40]. The structure of TLR-3 and TLR-8 receptor was downloaded from RCSB PDB database and the refined 3D structure of the multi-epitope construct was used as a ligand. Finally, the binding affinity between the TLR-3 and TLR-8 with vaccine construct was calculated by using H dock server (http://hdock.phys.hust.edu.cn/) and ClusPro 2.0 server (https://cluspro.bu.edu/login.php) [41]. The best docked complex was obtained based on the lowest energy weighted score and docking efficiency. The structure of the docked complex was visualized by the software PyMol. Afterwards, the docked complexes of vaccine with TLR3 and TLR8 respectively were subjected to 150 ns molecular dynamics (MD) simulations with Gromacs 2020.4 [42] package on HPC cluster at Bioinformatics Resources and Applications Facility (BRAF), C-DAC, Pune. The input topolologies of TLRs and vaccine were prepared using the CHARMM-36 force field [43,44]. The TLR-vaccine complexes were placed in a dodecahedron unit cell and solvated with TIP3P water model [45]. The solvated systems were neutralized with addition of suitable counter-ions, where TLR3-vaccine system needed addition of 4 chloride ions, while TLR8-vaccine system needed one sodium ion. The neutralized systems were energy minimized with the steepest descent algorithm until the force constant reaches 1000 KJ mol^-1^ nm^-1^. The systems were then equilibrated for 1 ns each at constant volume and constant temperature conditions (NVT) where the temperature of 300 K was achieved with modified Berendsen thermostat [46], and at constant volume and constant pressure (NPT) conditions where the pressure of 1 atm was achieved with Berendsen barostat [47]. The production phase MD simulations of 150 ns were performed on each equilibrated system with modified Berendensen thermostat and Parrinello-Rahman barostat [48] and restrains on covalent bonds with the LINCS algorithm [49]. The long range electrostatic energies were computed with Particle Mesh Ewald (PME) method [50] with the cut-off of 12 Å. Post MDS analysis was performed for root mean square deviations (RMSD) in C-α atoms, root mean square fluctuations (RMSF), and radius of gyration (Rg), where these analysis were performed for TLR chains and vaccine separately. The inter-chain hydrogen bond formation was also analyzed. The distance matrix was constructed for each system from mean smallest distance between residue pairs and contact maps were generated. The major paths of motion of each TLR chains and vaccine was analyzed through Principal Component (PC) analysis [51]. First two PCs were used in Gibb’s free energy landscape (Gibb’s FEL) analysis. The change in secondary structure of TLR chains and vaccine during simulation was also analyzed. The extent to which the fluctuations and displacements in side chains in each complex are correlated with one another was assessed from dynamic cross-correlation matrix (DCCM). The trajectories extracted at each 2 ns from 100 to 150 ns simulation period were subjected to Poisson Boltzmann surface area continuum solvation (MM-PBSA) calculations using g_mmpbsa program [52] to derive the binding free energy estimates between TLR chains and vaccine.

### 2.5. Disulfide engineering and *in silico* cloning studies

Vaccine protein disulfide engineering was performed using the Disulfide by Design 2 server (http://cptweb.cpt.wayne.edu/DbD2/) to investigate the conformational stability of folded proteins. Throughout the analysis, the Cα-Cβ-Sγ angle was kept at its default values of 114.6° ± 10 and the χ3 angle was set at -87° or +97°. Residue pairs with energies lower than 2.5 Kcal/mol were chosen and converted to cysteine residues in order to form disulfide bridges [53].

The vaccine construct 1 was cloned using *E*. *coli* strain K12 as the host. A codon adaptation tool JCAT (http://www.jcat.de) was utilized to adjust the codon usage to the well-characterized prokaryotic organisms in order to accelerate the expression rate in it since human and *E*. *coli* codon usage differs from each other. When utilizing the server, users should avoid the prokaryotic ribosome binding site, *BglII* and *Apa1* cleavage sites, and Rho independent transcription termination. The vaccine construct 1 optimal sequence was reversed, and then the N- and C-terminal *BglII* and Apa1 restriction sites were conjugated to it. Between the *BtgI* (2196) and *HincII* loci(181), the modified sequence was inserted into the pET28a(+) vector using the SnapGene restriction cloning module [54].

### 2.6 Immune simulation studies

The immunological simulation of the vaccine was performed using the C-ImmSim online server (http://150.146.2.1/C-IMMSIM/index.php), which provides a true immune interaction prediction [55]. The immune simulation left all parameters default, with the exception of time steps (set at 1, 84, and 170), and the number of simulation steps was set at 1,050. The vaccine’s recommended dosage is three injections every four weeks, which aligns with the appropriate time between doses for all commercial vaccines [56].

## 3. Result

### 3.1. Identification and Biophysical property analyses of the proteins

Similar proteins between monkeypox virus and smallpox virus were identified by using BlastP [21]. Where proteins of monkeypox virus were blast against the whole genome of smallpox virus. By analyzing BlastP identity percentage, five proteins were identified (S1 Table). The biophysical properties of the proteins are elucidated in Table 1. Among the similar proteins, two virulent proteins MPXVgp165 (Accession ID: UTG40861.1) and Virion core protein P4a (Accession ID: YP_010377118.1) were selected based on the antigenicity (>0.4), stability index, transmembrane topology, allergenicity, stability index and Grand average of hydropathicity (GRAVY) **(**Table 1). Both the selected proteins are virulent and core protein, previous study suggests that the virulent and core proteins could be effective vaccine target [57,58].

**Table 1:** Properties of selected similar proteins of monkeypox virus.

Protein	Antigenic score (Vaxijen)	Stability (ProtParam)	Topology (TMHMM)	Allergenicity (AllergenFP, AllerTOP)	Gravy (ProtParam)	Toxicity (Toxinpred)
>UTG40861.1 MPXVgp165 [Monkeypox virus]	0.4740	Stable	Outside	Non-allergen	-0.343	Non-toxic
>YP_010377118.1 Virion core protein P4a [Monkeypox virus]	0.4656	Stable	Outside	Non-allergen	-0.182	Non-toxic
>AIE40587.1 CD47-like putative membrane protein [Monkeypox virus]	0.4324	Stable	Outside/Inside	Non-allergen	0.696	Non-toxic
>UTG40742.1 MPXVgp048 [Monkeypox virus]	0.4236	Stable	Outside	Non-allergen	-0.127	Non-toxic
>USS79443.1 A5L [Monkeypox virus]	0.4019	Unstable	Outside	Non-allergen	-0.674	Non-toxic

### 3.2. Epitope mapping for peptide fusion

**Fig 2:**
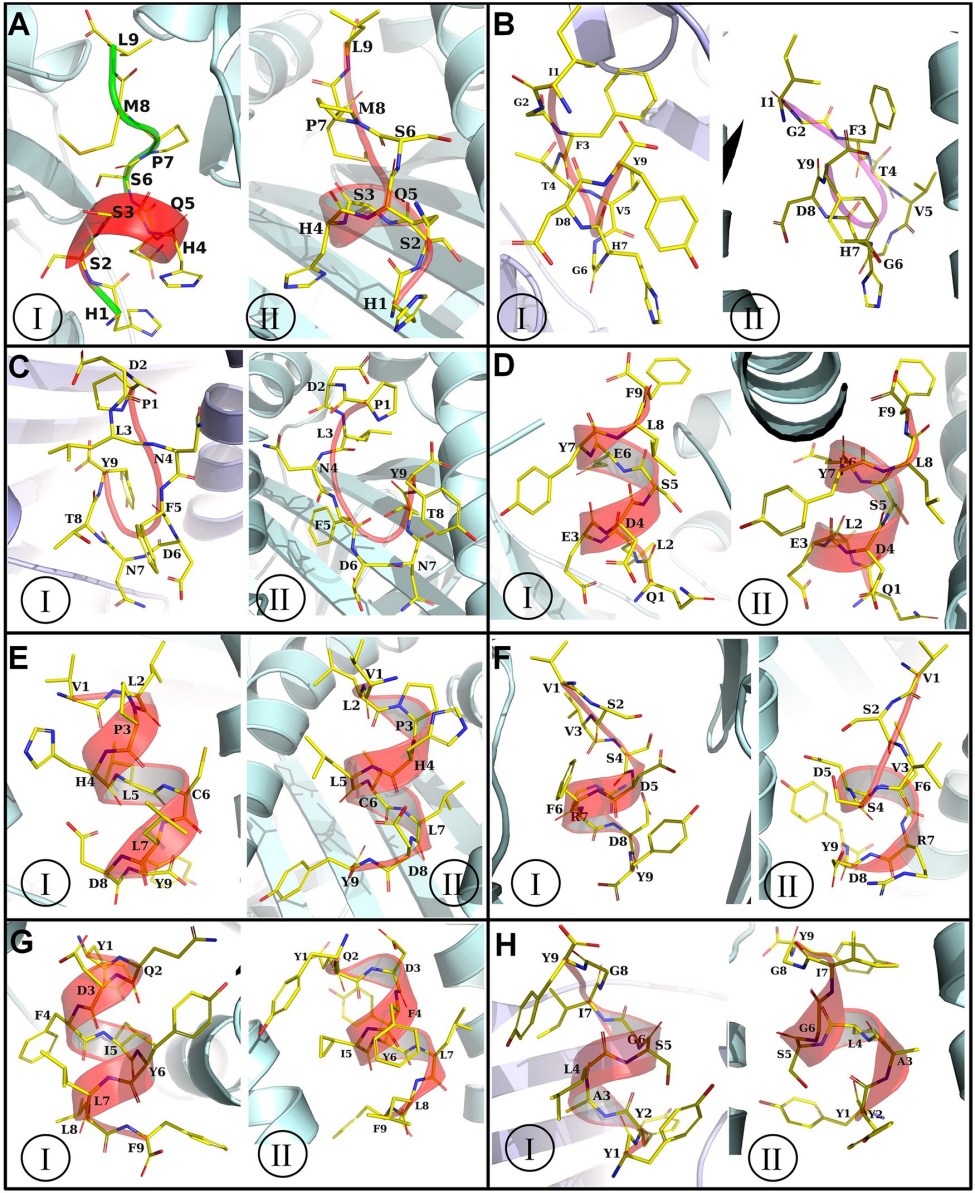
HLA alleles and epitope docked complexes. In each panel of figures, I is HLA-DRB 0401 allele and II is HLA-A1101 allele. Further, in each panel the docked epitopes are A) HSSHQSPML, B) IGFTVGHDY, C) PDLNFDNTY, D) QLEDSEYLF, E) VLPHLCLDY, F) VSVSDFRDY, G) YQDFIYLLF, and H) YYALSGIGY. (HLA allele is shown in light cyan color cartoon, epitopes are shown in semitransparent cartoon along with nontransparent yellow stick representation).

**Fig 3:**
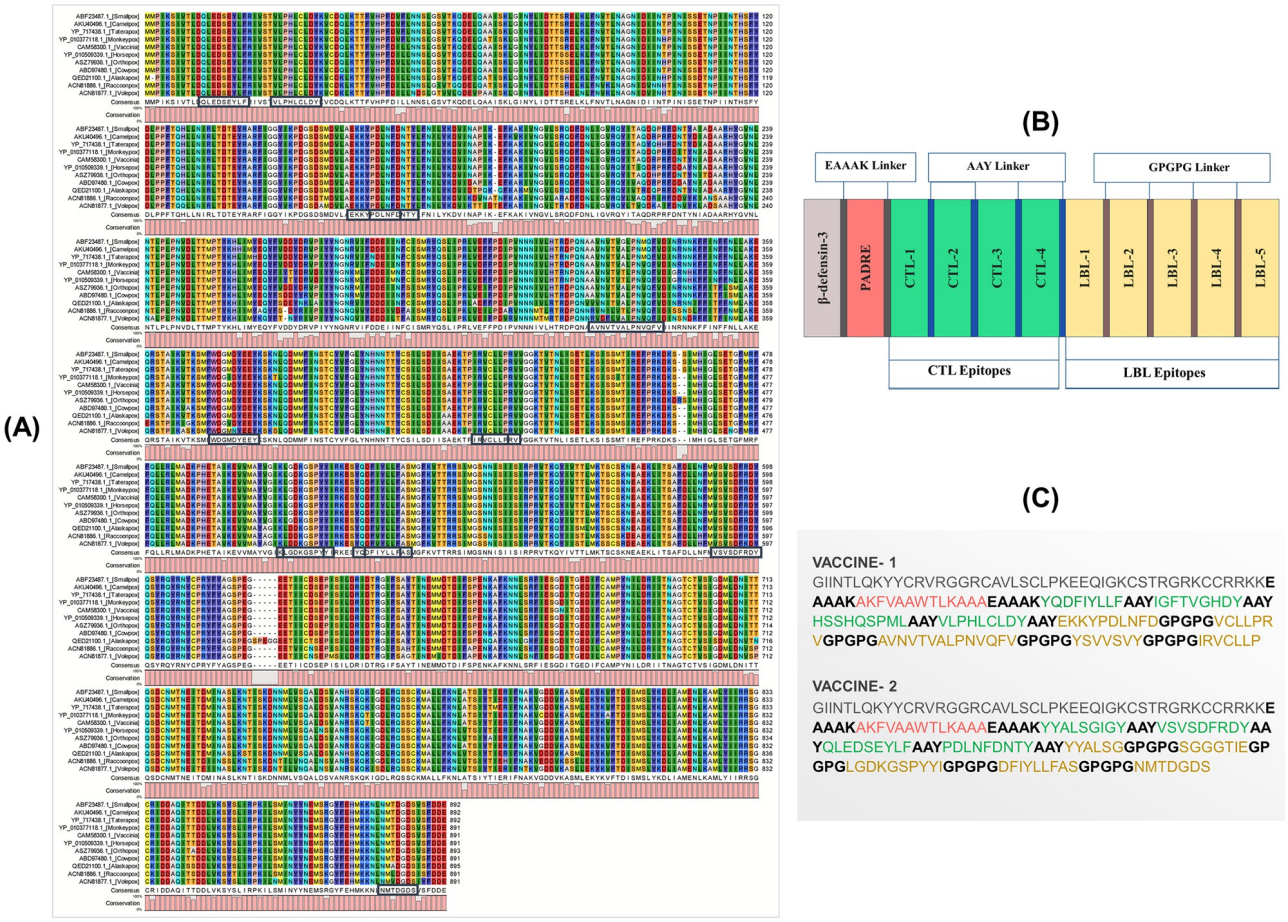
(A) Predicted conserved T-cell and B-cell epitopes within the Virion Core Protein P4A across different poxviruses (B) Schematic representation and (C) the sequence of the predicted vaccine constructs with associated linkers (EAAAK, AAY & GPGPG), adjuvant (human beta-defensin-3), PADRE sequence, and epitopes (T Cell & B Cell Epitopes).

According to default parameter settings, the NetCTL prediction method of IEDB server identified a total of 76 potential T cell epitopes [25]. The T cell epitopes were then tested and those that showed high antigenicity, non-toxicity, non-allergenicity, and conservation across populations were selected for further analysis. From these, the top 11 T cell epitopes (4 from MPXVgp165 and 7 from Virion core protein P4a) were chosen and their interacting MHC-1 alleles were identified (S2 Table). 3D structures were predicted to analyze their interactions with different HLA alleles. The majority of the putative T cell epitopes showed high binding affinity against both HLA-A*11:01 and HLA-DRB1*04:01 alleles when docking was performed by H-dock server [28]. Based on binding affinity and root mean square deviations (RMSD) value, the most promising T cell epitopes were selected as listed in Table 2. Their molecular interaction with both HLA alleles are shown in Fig 2. Additionally, six distinct B cell epitope prediction techniques of the IEDB server were used to predict top B cell epitopes (S3 Table). Ten B cell epitopes and eight T cell epitopes were finally selected for vaccine construction based on common stringent criteria, including high antigenicity, non-allergenicity, non-toxicity, and population-wide conservation, as listed in S4 Table. Furthermore, the conservancy of the epitopes predicted from the Virion core protein P4A was assessed using the CLC Drug Discovery Workbench 3 software version 3.0, which revealed that all the epitopes were conserved among different poxviruses [59]. Specifically, these epitopes were found to be fully conserved among monkeypox virus and some related *orthopoxviruses* including vaccinia, horsepox, taterapox, cowpox, alaskapox, camelpox, raccoonpox, orthopox, and volepox (Fig 3A).

**Table 2:** Docking score of selected T cell epitopes with HLA alleles.

Protein	Epitopes	Docking score	RMSD
HLA-A*11:01	YQDFIYLLF	-235.86	64.15
	IGFTVGHDY	-234.71	65.86
	HSSHQSPML	-219.41	48.19
	VLPHLCLDY	-218.60	48.87
	YYALSGIGY	-203.79	63.46
	VSVSDFRDY	-199.22	65.96
	QLEDSEYLF	-197.30	69.51
	WDGIDYEEY	-193.81	65.87
	PDLNFDNTY	-183.88	45.57
	MDSMEALEY	-172.35	63.41
	KLGDKGSPY	-145.65	63.88
HLA-DRB1*04:01	YQDFIYLLF	-236.39	54.67
	HSSHQSPML	-225.25	71.99
	VLPHLCLDY	-211.42	54.13
	YYALSGIGY	-211.38	62.16
	IGFTVGHDY	-193.80	69.32
	VSVSDFRDY	-192.10	73.78
	QLEDSEYLF	-186.07	70.14
	PDLNFDNTY	-182.20	94.97
	WDGIDYEEY	-168.90	62.98
	KLGDKGSPY	-162.90	56.64
	MDSMEALEY	-159.66	53.16

### 3.3. Analyses of the biophysical and structural properties of the constructed vaccine

**Fig 4:**
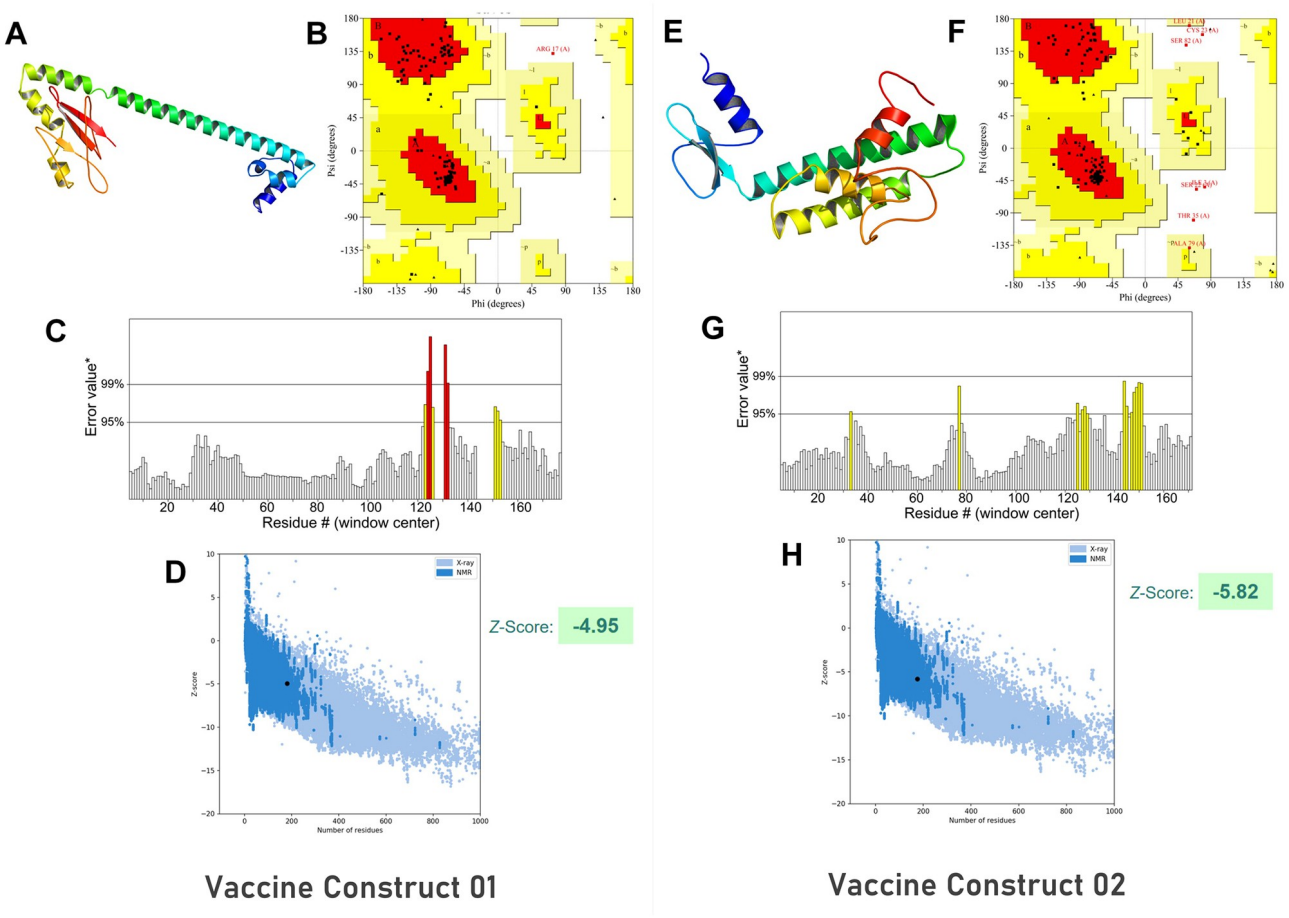
Tertiary structure prediction and validation of the vaccine constructs A) 3D model, B) Ramachandran plot, C) Errat plot and D) Z-Score showing quality aspects of vaccine construct 1 and E) 3D model, F) Ramachandran plot, G) Errat plot and H) Z-Score showing quality aspects of vaccine construct 2.

Specific linkers and adjuvants were used to conjugate the epitopes for vaccine construction as shown in schematic and constructive representations in Fig 3B and 3C, respectively. Two vaccine constructs were designed, each containing four T cell epitopes and five B cell epitopes. Rigorous testing was performed to ensure high antigenicity, non-allergenicity, and non-toxicity of the vaccine constructs, with biophysical analyses demonstrating favorable properties such as solubility, stability, and suitability for additional analyses (S5 Table). Secondary structure analysis of the vaccine constructs revealed random coil as the most dominant structure. The trRosetta web server was used to predict the 3D structure of the vaccine constructs, which were subsequently refined and validated. The Ramachandran plot demonstrated that a majority of the residues were in the favored region for both vaccine constructs, with 95.2% of all residues in the favored region for vaccine construct 1 and 86.1% for vaccine construct 2. The 3D model and validation of both vaccine constructs are depicted in Fig 4. S6 Table, S1 Fig provide additional details on the secondary structure of the vaccine constructs. The discontinuous B cell epitope residues for both vaccine constructs were listed in S7 Table.

### 3.4. Molecular docking and dynamics simulation studies

Molecular Docking analysis was conducted using H dock and ClusPro server and molecular dynamic simulation was performed through Gromacs 2020.4 package to evaluate the interaction and binding affinity of vaccine construct with toll-like receptor 3 (TLR3) and toll-like receptor 8 (TLR8) [42]. The results indicated that Vaccine construct 1 exhibited a significantly higher free binding energy and demonstrated a greater docking score with both TLR3 and TLR8. The docking scores for both H dock and ClusPro server are presented in S8 Table. Subsequently, based on the assigned docking score, the vaccine construct 1 was chosen to undergo additional Molecular Dynamics (MD) simulations for further analysis.

**Fig 5:**
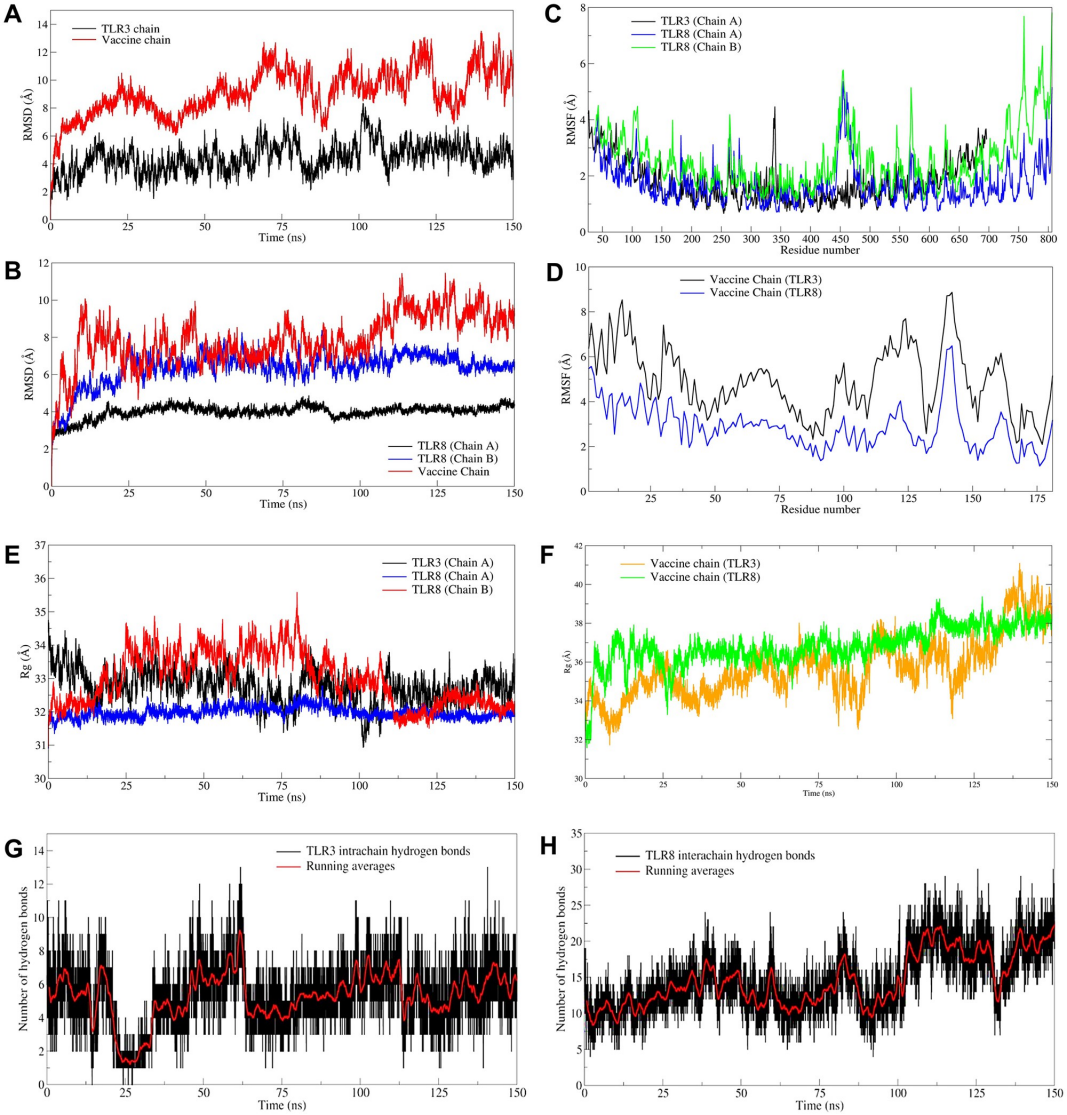
The RMSD, RMSF, Rg, and Hydrogen bond analysis plots. RMSD against simulation time plot for A) TLR3 chain and bound vaccine chain, and B) TLR8 chains and bound vaccine chain, RMSF in side chain atoms of residues of C) TLR3 and TLR8 chains, and D) Vaccine chain, total Rg plotted against simulation time in E) TLR chains, F) vaccine chain, and Number of hydrogen bonds formed between G) TLR3 chain A and vaccine chain during MD simulation, and H) TLR8 chains (A and B) and vaccine chain during MD simulation.

The docked complexes of vaccine with TLR3 and TLR8 respectively were subjected to 150 ns MD simulations. In the case of TLR3-vaccine complex, the RMSD in TLR3 chain is stable compared to the corresponding vaccine chain (Fig 5A). The RMSD in TLR3 chain is below 6 Å until around 100 ns and thereafter for a brief period of around 10 ns deviates reaching the maximum extent of deviation to 8.33 Å and thereafter remains stable till the end of simulation period (Table 3). While, the RMSD in C-α atoms of vaccine bound to TLR3 has higher magnitude of deviations reaching maximum of 14.947 Å. In the case of TLR8-vaccine complex the RMSD in C-α atoms of chain A is lower than chain B (Fig 5B). The RMSD in C-α atom of chain A is quite stable with an average of 3.994 Å. On the other hand, the RMSD in chain B is slightly higher with an average 6.367 Å. The RMSD in bound vaccine has major deviations until 100 ns simulation period, and thereafter remains stable with an average of 7.873 Å. Comparably the RMSD in vaccine bound to TLR8 is lower than the vaccine bound to TLR3.

**Table 3:** Average, minimum and maximum values of different MDS analysis parameters.

	TLR3 (chain A)	Vaccine chain (bound to TLR3)	TLR8 (Chain A)	TLR8 (Chain B)	Vaccine chain (bound to TLR8)
**RMSD (Å) in C-α atoms**					
Minimum	0.869	1.457	1.351	1.462	1.096
Maximum	8.333	14.947	4.856	8.365	11.444
**RMSF (Å)**					
Average	1.756 (0.732)	4.939 (1.493)	1.725 (7.466)	2.526 (1.046)	2.870 (0.988)
Minimum	0.668	2.098	0.715	1.110	1.138
Maximum	4.460	8.865	5.361	7.808	6.485
**Rg (Å)**					
Average	32.753 (0.351)	35.727 (0.834)	31.984 (0.095)	32.993 (0.493)	36.831 (0.806)
Minimum	30.939	31.737	30.914	30.963	31.5593
Maximum	34.739	41.086	32.775	35.578	39.360

Standard deviations in average values are given in parentheses.

The results of Root mean square fluctuation (RMSF) analysis showed that the major fluctuation of 4.446 Å occurred in side chain atoms of residue Leu340 in TLR3 chain (Fig 5C). Most of the other residues in the range 150 to 600 showed RMSF of around 2 Å. The RMSF in TLR8 chain A showed major fluctuation in side chain atoms of residues in the range 448–467 reaching maximum RMSF of 5.361 Å in residue Arg455. Compared to RMSF in the TLR8 chain A, the RMSF in chain B residues is slightly higher. In TLR8 chain B the major fluctuations were observed in residues in the range 447–482. The vaccine bound to TLR 8 showed lower RMSF compared to vaccine bound to TLR3 (Fig 5D).

**Fig 6:**
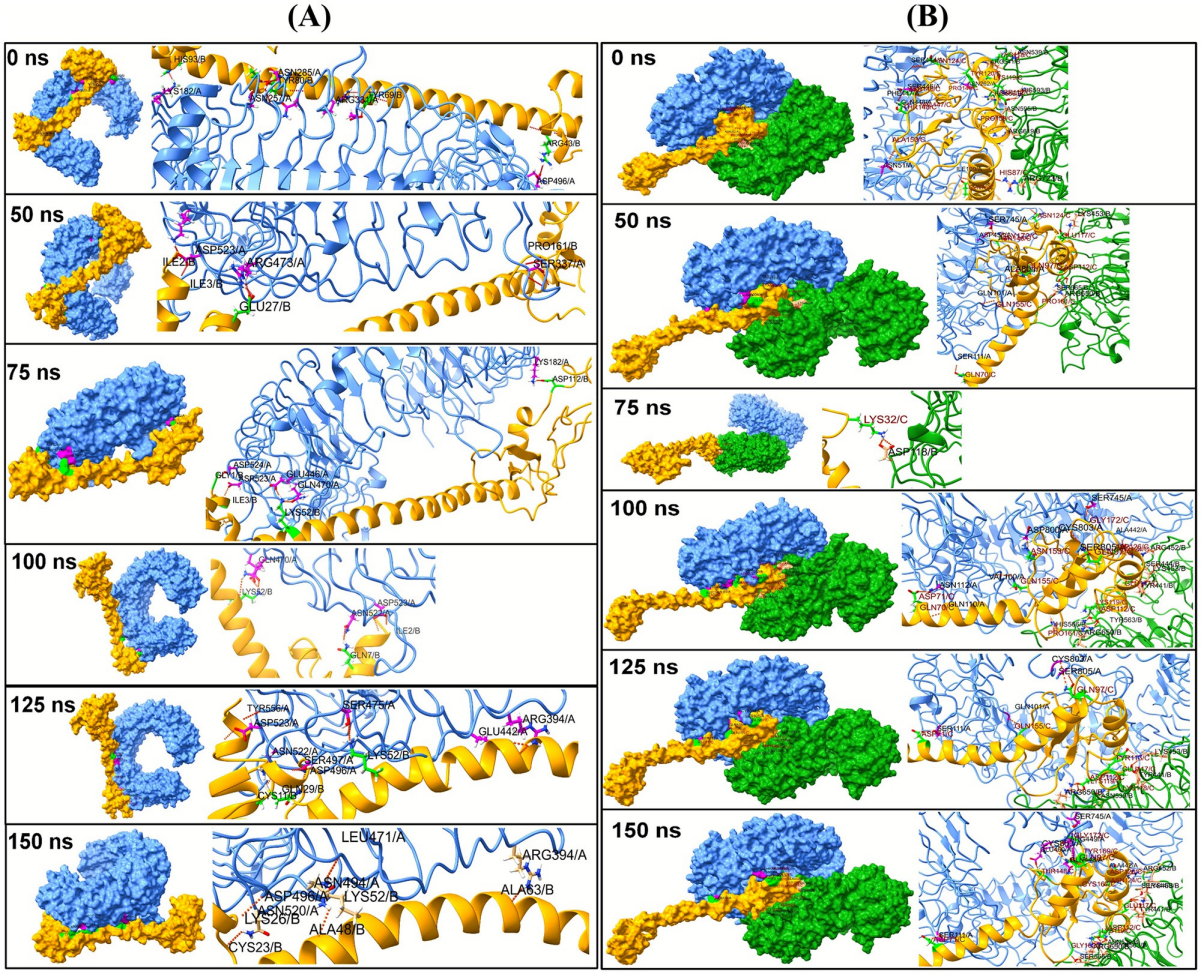
(A) The inter-chain hydrogen bonds between TLR3 chain A and vaccine chain B (B) The inter-chain hydrogen bonds between TLR8 chain A, chain B and vaccine chain C. Surface representations of TLR3 chain A and vaccine chain, are shown in light blue and orange colors, respectively, with corresponding cartoon representations. Interacting residues of TLR3 are in pink and green is for the vaccine. Similarly, TLR8 chain A, chain B, and vaccine chain C are shown in light blue, green, and orange colors, respectively. Interacting residues of TLR8 chain A are in pink, light brown represents chain B, and green is for the vaccine.

**Fig 7:**
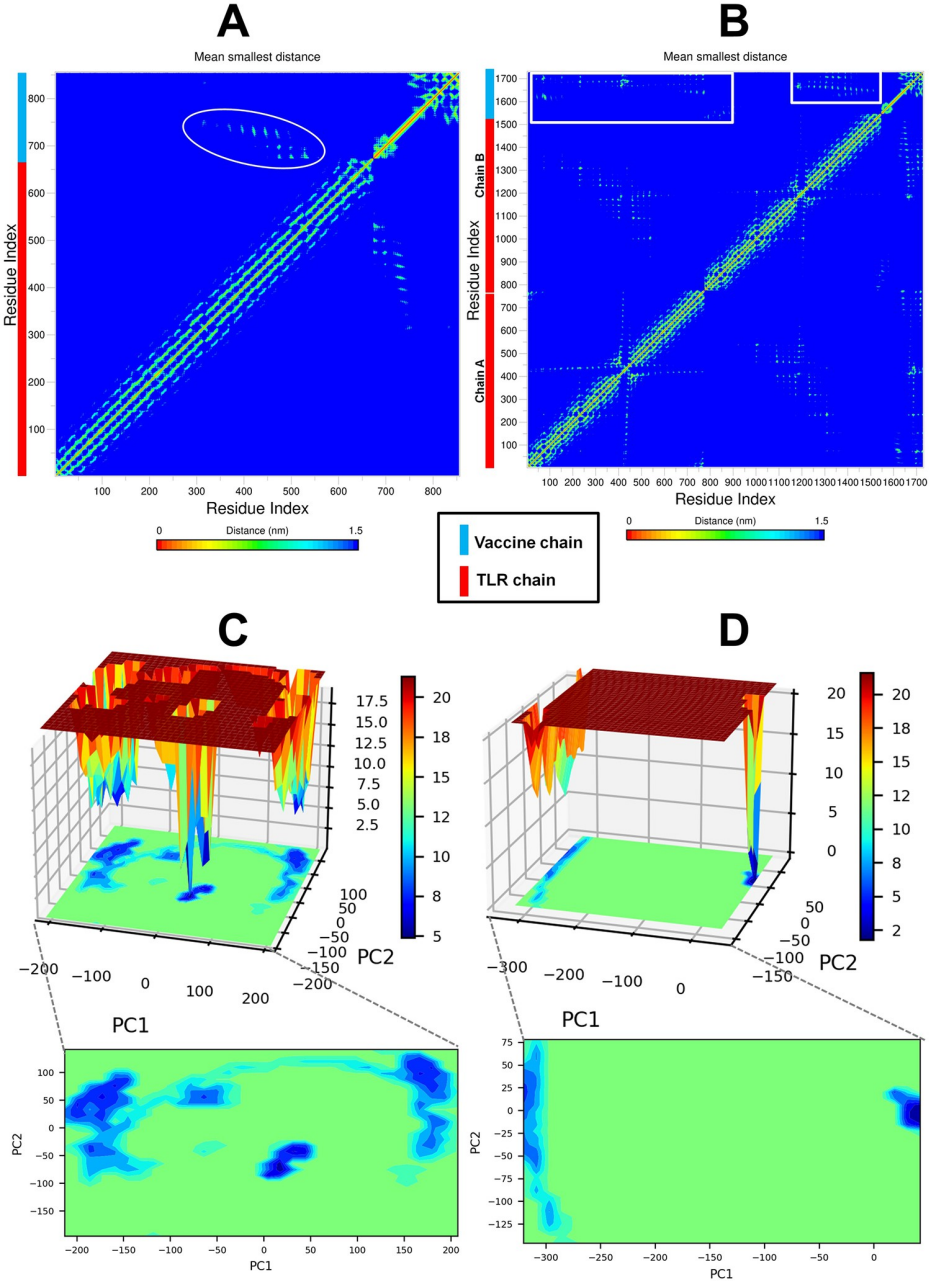
Contact maps constructed on mean smallest distances between network of residues and Gibb’s free energy landscapes. Contact map of (A) TLR3-vaccine complex, and (B) TLR8-vaccine complex (The interchain residue-residue contacts are marked shown in white circle and white rectangles in TLR3 and TLR8-vaccine complexes respectively) and Gibb’s free energy landscape of (C) TLR3-vaccine complex, and (D) TLR8-vaccine complex.

**Fig 8:**
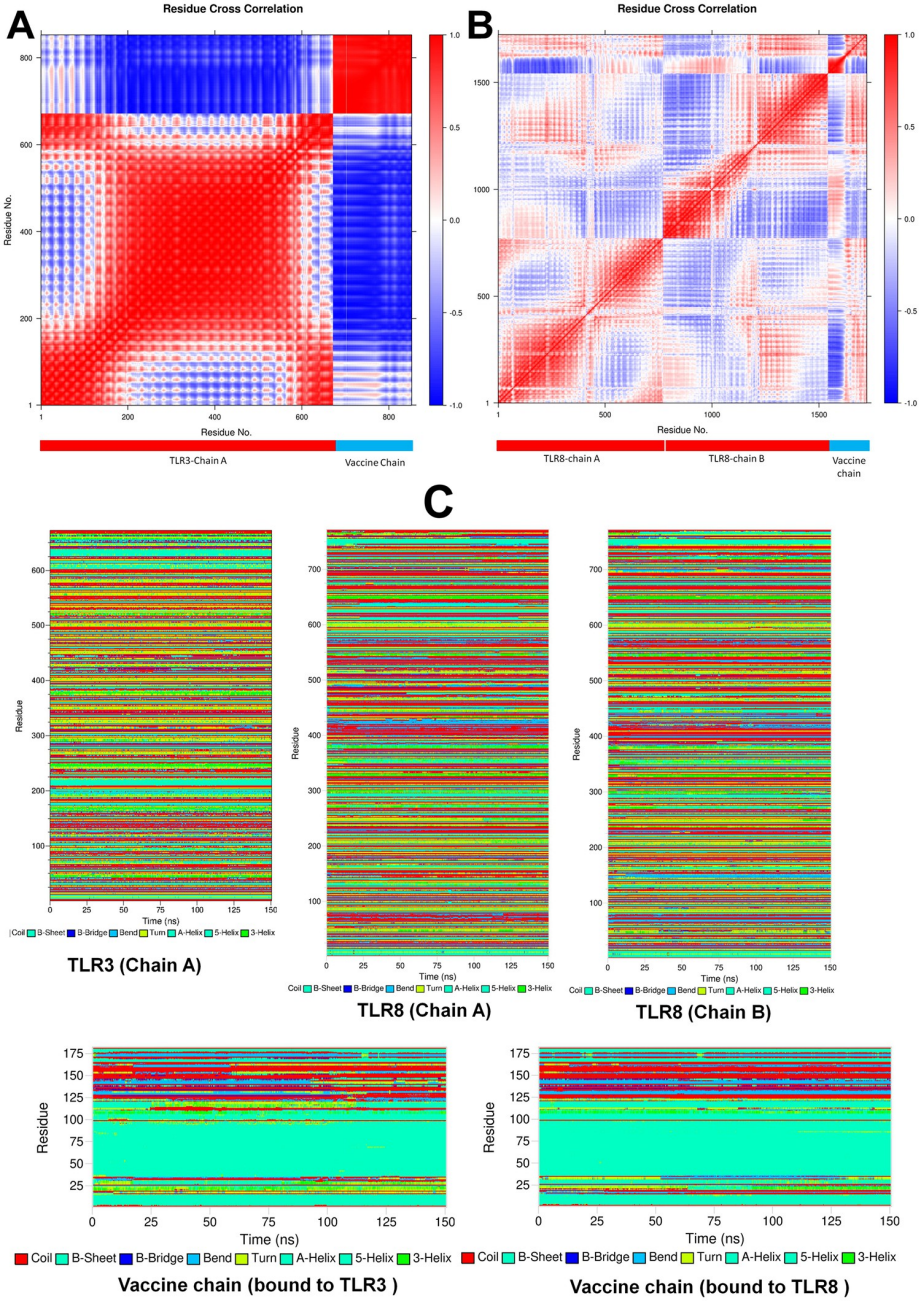
DCC analysis for vaccine chain complex with A) TLR3, and B) TLR8. (The plot of DCCM shows residue-wise correlation in each complex. The separation of TLR chain and vaccine chain is shown at the bottom of each plot.) (C) DSSP plots for individual TLR chains and corresponding vaccine chain in the complex.

The results of radius of gyration (Rg) of TLR3 and TLR8 chains showed that the chain A of TLR3 has higher Rg than the chain B of TLR8 with an average Rg of 32.753 Å and 32.993 Å respectively (Fig 5E). The overall average Rg for chain A of TLR8 is 31.984 Å which is stable compared to chain A of TLR3 and chain B of TLR8. The Rg of vaccine chain bound to TLR8 is quite stable with an average Rg of 36.831 Å (Fig 5F). The vaccine chain bound to TLR3 showed the deviations in the Rg throughout the simulation period with an average of 35.753 Å. The interchain hydrogen bonds formed between TLR chain and vaccine was analyzed. In the case of TLR8-vaccine complex the vaccine is bound at the interface of two chains. Thus, the hydrogen bonds formed between TLR3 chain A and vaccine, and hydrogen bonds between TLR8 chain A/B and vaccine were analyzed (Fig 5). The results showed that more number of interchain hydrogen bonds formed in TLR8-vaccine complex than TLR3-vaccine complex. Specifically, the average number of hydrogen bonds formed per timeframe in between TLR3 chain A-vaccine chain is 5.629; while the average number of hydrogen bonds formed per timeframe between TLR8 chain A/B-vaccine chain is 14.716. In the case of TLR3-vaccine complex, average around 6 hydrogen bonds were formed (Fig 5G). While, in the case of TLR8-vaccine complex, around 7 hydrogen bonds were formed (Fig 5H). More than 15 average number of hydrogen bonds between TLR8 chain A/B together and vaccine chain. The trajectories at 50, 75, 100, 125 and 150 ns were visually inspected. The initial trajectory of TLR3-vaccine complex showed the hydrogen bonds between Lys182, Asn257, Asn285, Arg331, Asp496 residues of TLR3 chain and His93, Tyr80, Tyr69, and Arg43 residues of vaccine initially (Fig 6A). However, all these hydrogen bonds are seemingly weaker and transient and the equilibrated trajectory at end of simulation showed the hydrogen bonds between Arg394, Asn494, Asn520, Asp496, Leu471 residues of TLR3 chain and Lys26, Lys52, Ala63, Ala48, and Cys23 of vaccine chain. The analysis of TLR8-vaccine complex showed that the vaccine remained bound at the interface of TLR8 chain A and B except at around 75 ns period (Fig 6B). The initial trajectory of TLR8-vaccine complex showed the existence of 18 hydrogen bonds, where the residues Gln70, Lys118, Lys119, Tyr120, Asn124, Val147, Thr148, Ala150, Pro140, Asn146, Asp112, Pro159, His87 of vaccine make hydrogen bond with Asn51, Ile109, Asn262, Phe447, Gln448, Ile109, Ser444, and Ser446 residues of chain A and Arg541, His593, Asn595, Arg619, Arg723, Asn539, His566, and His593 residues of chain B. The last equilibrated trajectory at 150 ns trajectory showed the hydrogen bond between Thr148, Gly162, Tyr169, Gly172, Asn124, Cys167, Tyr169, Asp126 residues of vaccine chain and Ser111, Ala442, Gln448, Arg449, Ser745, and Glu460 residues of chain A and Tyr441, Ser444, Arg452, Tyr563, and Ser565 residues of chain B. The extent of residue-residue contact between respective TLR chain and vaccine was analyzed through contact map analysis (Fig 7). Within the vaccine chain in both the complexes, the region constituting the α-helices (residue 37–97) are devoid of any residue-residue contacts, while the terminal parts of vaccine chain and specifically the region comprising residue 98–181 have the intra-chain residue-residue contacts. Comparatively fewer number of intra-chain residue contacts are seen in TLR3-vaccine complex than TLR8-vaccine complex (Fig 7A and 7B). The stability of the corresponding systems was studied through Principal Component (PC) and Gibb’s free energy analysis. The lowest energy basins were found with TLR8-vaccine complex compared to TLR3-vaccine complex. Specifically, out of two energy basins for TLR8-vaccine complex, the one with energy range -350 to -300 kJ mol^-1^ on PC1 and -75 to 50 kJ mol^-1^ on PC2 is largest energy basin (Fig 7D). While, for TLR3-vaccine complex the lowest energy basin was observed at energy range -200 to -150 kJ mol^-1^ on PC1 and -50 to 100 kJ mol^-1^ on PC2 **(**Fig 7C). The results of Dynamic Cross Correlation Matrix (DCCM) analysis indicate that the TLR3-vaccine complex has fewer moderately correlated residues in the range residue 1 to 100 of chain A with 1 to 97 residues of vaccine (Fig 8A and 8B). On the other hand, the TLR8-vaccine chain show many positively correlated residue-residue crosswalks between chain A and vaccine and slightly stronger positively correlated residue-residue crosswalks between TLR8 chain B and vaccine (Fig 8A and 8B).

The results of Definition of secondary structure of proteins (DSSP) analysis showed that the TLR3 chain A remained quite stable throughout the MD simulation (Fig 8C); while the secondary structural change is evident in TLR8 chains in the residues ranging from 400 to 450 and terminal residues beyond 750. The α-helices ranging from residue 37 to 97 in vaccine bound to both the TLRs remained quite unaltered. The results of MM-PBSA calculations are given in Table 4 which clearly show that the TLR8-vaccine complex has favorable binding energy compared to TLR3-vaccine complex. Except polar salvation energy TLR8-vaccine complex has all the individual energy contributions more favorable compared to TLR3-vaccine complex.

**Table 4:** Results of MM-PBSA calculations.

Complex with vaccine	van der Waal energy (kJ mol^-1^)	Electrostatic energy (kJ mol^-1^)	Polar solvation energy (kJ mol^-1^)	Solvent accessible surface area energy (kJ mol^-1^)	Binding energy (ΔG_binding_) (kJ mol^-1^)
TLR3	-0.017 (0.013)	-164.942 (2.687)	-15.071 (1.839)	1.357 (0.023)	-178.673 (3.999)
TLR8	-1010.465 (3.681)	-887.143 (3.831)	1458.195 (10.294)	-131.192 (0.449)	-571.404 (7.134)

### 3.5. Disulfide engineering and *in silico* cloning studies

By using the DbD2 server, a total of 13 pairs of amino acid residue for vaccine construct 1 have been discovered as having the ability to structure disulfide bonds and two pair (Val 132—Ser 167 and Pro 152 –Gly 170) were selected that are convenient to the standards for disulfide bond formation (S2 Fig).

The protein expression systems for humans and *E*. *coli* are different, this is why codon adaptation was performed. Reverse transcription was used for the vaccine construct 1. The greater proportion of most abundant codons was shown by the codon adaptation index (CAI) of the adapted codons. The optimized codons’ GC content (50.73%) and CAI (0.89015) were both shown to be significant. The construct’s safety for cloning was guaranteed by the absence of restriction sites for *BtgI* and *HincII*. The optimized codons, along with *BtgI* and *HincII* restriction sites, were inserted into the pET28a (+) vector. A clone of 3684 base pairs was generated, which included the desired sequence of 330 bp and the remainder belong to the vector. Red was used to indicate the desired area between the pET28a (+) vector sequence. (S3 Fig).

### 3.6 Immune simulation studies

**Fig 9:**
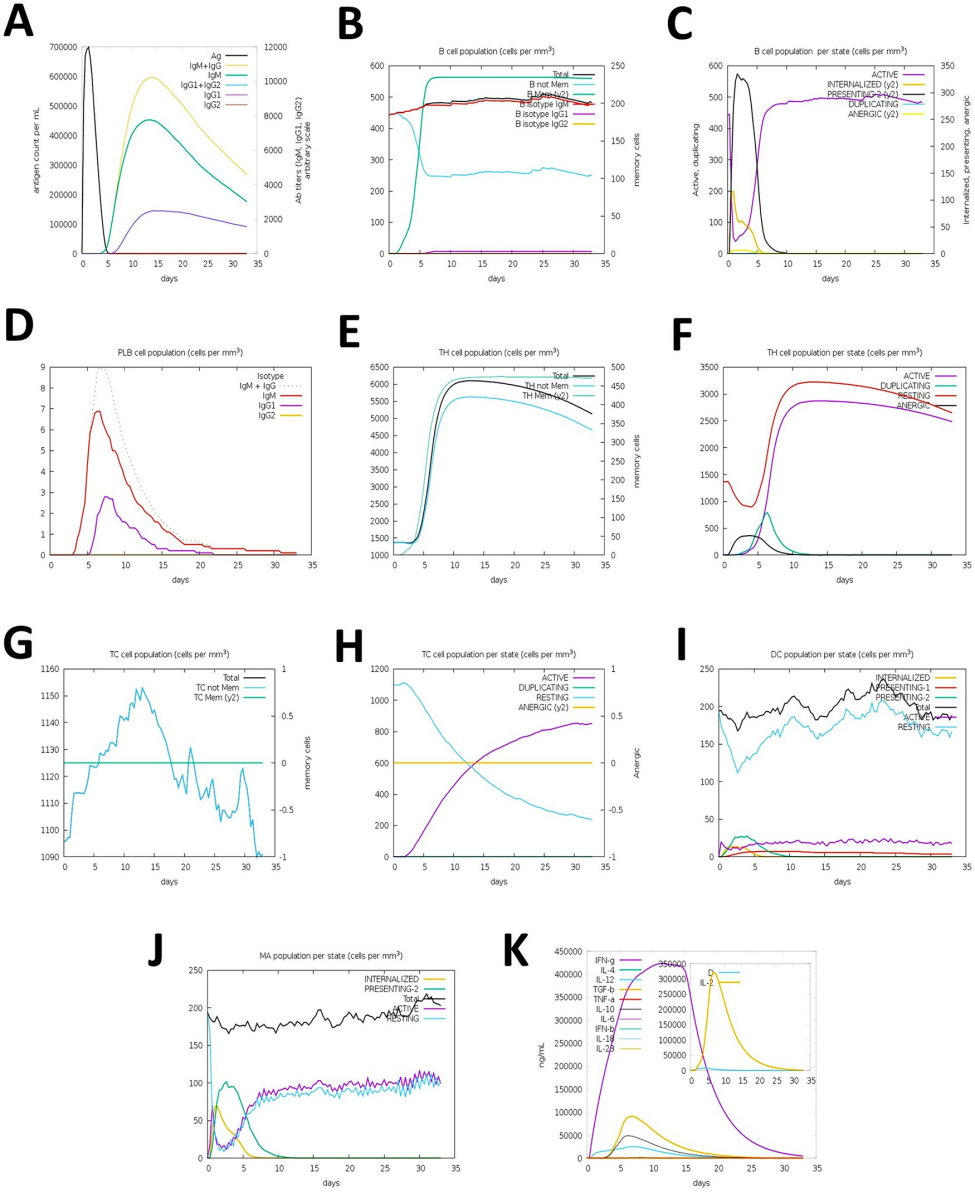
C-ImmSimm represents the immunological stimulation of the best predicted vaccine. (A) Black line represents the immunoglobulin and immunocomplex responses to vaccine immunisations and the subclasses are denoted by coloured lines (B) Elevation in the B-cell population (C) Inclination of the B-cell population by state during vaccination. (D) An upsurge in plasma B-cell (PLB) population over the course of the injections. (E) The helper T-cell (TH) population grew during the course of three injections. (F) Enhancement in the helper T-cell population per state throughout vaccination. (G) Increased regulatory T lymphocyte (TC) activity across the course of three injections. (H) The cytotoxic T lymphocyte population rose throughout the infusions. (I) Increase in the active cytotoxic T lymphocyte population per state throughout the course of three injections; DC, dendritic cell. (J) The active dendritic cell population increased in each state during the three injections: MP and macrophages. (K) The concentrations of many cytokines elevated throughout the course of three dosages.

The immunological simulation study revealed that the vaccination has the ability to elicit a typical immune response that is consistent with the natural immune system. The vaccine was projected to strongly activate primary immune responses following each of the three administrations sequentially. Furthermore, the secondary immune response was activated, whereas the primary immune response progressively grew with each dose. In addition, subsequent increases in the concentrations of active B cells, plasma B cells, helper T cells, and cytotoxic T cells were discovered, indicating the generation of an exceedingly robust immune response and memory of immunity, as well as higher antigen clearance in the host. The vaccine has the potential to induce a broad spectrum of cytokines necessary for immune response and viral defense, including IFN-γ, IL-23, IL-10, and IFN-β. The result of immune simulation study is summarized and depicted in Fig 9. In brief, immune simulation studies predicted a number of promising vaccine properties, including the production of a large number of immunoglobulins, APCs, cytokines, and active B and T cells, implying that the polyvalent vaccine may generate outstanding immunological responses following administration within the host.

## 4. Discussion

The significant impacts of monkeypox virus and smallpox viruses on public health, including the potential for severe illness and death, underscore the crucial need for effective prevention and treatment strategies [59,60]. Utilizing a reverse vaccinology approach, the study aimed to retrieve conserved epitopes for these viruses in order to potentially provide cross-protection against related viruses with similar antigenic properties [61,62]. Additionally, investigating the molecular interactions between the vaccine and TLRs holds important implications for future research on preventing and managing infections or pandemics caused by monkeypox virus and related viruses. *In silico* epitope based vaccine were previously studied for different pathogenic organisms [63–68]. Two virulent proteins of monkeypox virus MPXVgp165 and Virion core protein P4a, were selected based on their similarity between targeted species, stability, and antigenicity. Virion Core Protein P4a is a vital component of poxvirus virulence and is essential for evading host immune responses [60] whereas, MPXV 165 protein can interfere with the function of dendritic cells, which are important for initiation and regulation of the immune response [69]. Both proteins were found to be antigenic and thus were subjected to epitope mapping for vaccine construction. The study focused on identifying both T cell epitope and B cell epitopes since both are important to strongly stimulate the host’s immune system in response to viral infection [70,71]. The epitopes were predicted using IEDB algorithms however, predicted T cell epitopes were further crosschecked through docking analysis with specific HLA alleles. Binding affinity of the selected epitopes was considered against HLA-A*11:01 and HLA-DRB1*04:01 alleles as these are two of the most commonly found receptor in human population [27]. Both the representative alleles can play significant role in presenting virus antigens to T cells and inducing an effective immune response [72]. All the selected epitopes displayed low binding energy which is biologically important. Epitopes that were finally selected for vaccine construction were highly antigenic, non-allergenic and non-toxic suggesting their suitability to induce good immune response against the viral infection. Selected T cell epitopes and B cell epitopes were finally combined using various linkers to ensure that the epitopes were sufficiently separated while constructing the vaccine. To enhance immunogenicity in the human body, PADRE and human beta-defensins adjuvants were conjugated in the vaccine sequence [73,74]. Two vaccines were constructed by utilizing the selected T cell epitopes and B cell epitopes. Both the vaccine construct were found to be highly antigenic, non-allergenic, nontoxic and soluble ensuring their potential to generate adequate immune response and to be safe for human body. Vaccine construct 1 has greater solubility than vaccine construct 2. Both constructs are stable and have a dominant random coil secondary structure. Tertiary structure prediction and validation show that vaccine construct 1 has a better structure profile than vaccine construct 2, with higher ERRAT score (94.444 vs 92.121) and more structure in the favored region (95.2% vs 86.1%). In molecular docking analysis, both vaccine constructs were docked with TLR3 and TLR8, showing good binding affinity in both cases. TLR3 recognizes dsRNA in some poxviruses, activating TRIF-mediated signaling to produce inflammatory cytokines and IFNs. TLR8 in myeloid cells can recognize poxviral RNA or DNA, inducing IFN production and proinflammatory responses [75] Vaccine construct 1 had the lowest docking score with both TLR3 and TLR8 (-1061.8 and -1176.6 respectively) in ClusPro server. Vaccine construct 2 also had good binding affinity however, based on solubility, physicochemical properties, and binding affinity, vaccine construct 1 was selected for molecular dynamic simulation studies.

The simulation studies revealed that the TLR3 chain A has a higher RMSD than the TLR8 chains, suggesting better stability of the TLR8-vaccine chain. The lower RMSD in the TLR8 bound vaccine, being stable and lower than the TLR3 bound vaccine, suggests the stability of the TLR8 bound vaccine. The lower RMSD in the TLR8 bound vaccine may be attributed to more consistent hydrogen bonds. Furthermore, the conformational motions of the TLR8 bound vaccine are restricted within the conformational space of two chains, which holds the vaccine tightly between these chains. This can be the reason for the lower RMSD compared to the conformationally free TLR3 bound vaccine. The RMSF in TLR3 chain A is quite stable but the vaccine bound to it has higher magnitude of RMSF. While, the chain B of TLR8 having slightly higher RMSF than chain A suggests the conformational changes in chain B residues (440 to 490). However, the vaccine bound at the interface of the two chains of TLR8 has comparably less RMSF than the vaccine bound to TLR3. This clearly suggests the stability of TLR8-vaccine complex over TLR3-vaccine complex. Although the Rg of TLR8 chain B is slightly higher than chain A which suggest the change in compactness of only chain B. Comparably chain A of TLR3 has larger magnitude of Rg indicating consistent change in the compactness of system. The Rg of vaccine bound to TLR8 being almost stable and reasonably converged after 25 ns indicating overall stability of TLR8-vaccine complex. While, the vaccine chain bound to TLR3 showing deviations throughout the simulation suggests a possible loss in the compact nature of it. The hydrogen bond analysis revealed more than 20 hydrogen bonds post 100 ns between chains of TLR8 and vaccine suggesting quite strong binding of vaccine with TLR8. Comparatively the fewer number of hydrogen bonds *i*.*e*. around 6 between vaccine and TLR3 suggests slightly weaker vaccine affinity. The positioning of vaccine chain between two chains of TLR8 has profound effect in formation of few stable hydrogen bonds and overall stability of TLR8-vaccine chain. The contact analysis suggested that the TLR8-vaccine complex has a greater number of residue-residue contacts between vaccine chain and both the chains of TLR8. Further, quite larger region of chain A residues of TLR8 found establishing key contacts with vaccine residues compared to chain B suggesting stability of TLR8-vaccine complex due to these interactions. It is evident from contact analysis that only few residues of TLR3 chain between the ranges 300–550 could establish key contacts with vaccine residues. Overall, the contact analysis suggested that TLR8-vaccine complex has good number of favorable residue-residue contacts. The Gibb’s free energy evaluation revealed the existence of more favorable low energy conformations of TLR8-vaccine complex compared to TLR3-vaccine complex. The DCCM analysis suggests that the TLR3-vaccine chain has very few positively correlated residue-residue crosswalks between TLR3 chain and vaccine, compared to TLR8-vaccine complex. The DCC analysis clearly suggested better association of residue-residue associations in TLR8-vaccine complex. Further, the DSSP analysis suggests no major secondary structural changes in either TLR3 chain A or the chains of TLR8. However, the residues in the range 400–450 in chain B of TLR8 showed secondary structural changes, which may be due to the interaction of vaccine with the residues in this range. In the case of vaccine major secondary structural changes are evident in the terminal domains except the α-helical region. More of such secondary structural changes are seen in vaccine bound to TLR3 suggesting slightly less favorable and less stable conformation of bound vaccine. Finally, the MM-PBSA analysis revealed that TLR8-vaccine complex having the more favorable binding affinity compared to TLR3-vaccine complex. Particularly, the van der Waal energy, electrostatic energy, solvent accessible energy is more favorable in the case of TLR8-vaccine complex. The findings suggest that TLR8 could be a more favorable target for vaccine development, as it forms a more stable and stronger complex with the vaccine compared to TLR3. This information can help researchers in designing more effective vaccines with improved stability and binding affinity. In conclusion, this study successfully retrieved conserved epitopes and provided molecular insights into the generation of the immune response, which will aid in future research for the design of polyvalent vaccines against monkeypox virus, smallpox virus, and related poxviruses.

## 5. Conclusion

Our study addresses the pressing public health concerns posed by monkeypox and smallpox viruses, aiming to design effective preventive strategies. Through reverse vaccinology, we identified conserved epitopes for potential cross-protection against related viruses. Molecular dynamics simulations revealed promising interactions between our vaccine constructs and TLRs, suggesting avenues for future vaccine development. However, limitations include the reliance on computational models and the need for further validation through experimental studies. Despite these challenges, our findings offer valuable insights into immune response generation and pave the way for the development of polyvalent vaccines against poxviruses, with the potential to mitigate the impact of future outbreaks.

## Supporting information

S1 Fig Predicted secondary structures of (A) Vaccine Construct 1 and (B) Vaccine Construct 2 using PSIPRED. (DOCX)

S2 Fig Disulfide engineering study of the vaccine construct 1 (A) Original model (B) Mutated model. (DOCX)

S3 Fig The recombinant plasmid designed for mass production of the proposed vaccine. The red-colored portion represents the constructed vaccine.
(DOCX)

S1 Table Similar proteins among Monkeypox virus and Variola virus (smallpox). (DOCX)

S2 Table List of Potential T cell epitopes with their antigenic score, allergenecity, toxicity, transmembrane topology and conservancy analysis with their interacting MHC-1 alleles. (DOCX)

S3 Table List of Potential B cell epitopes with their antigenic score, allergenecity, toxicity, transmembrane topology, conservancy analysis. (DOCX)

S4 Table List of the epitopes selected for vaccine construction (selection criteria: Docking score, antigenicity, nonallergenicity, transmembrane topology, nontoxicity and conservancy). (DOCX)

S5 Table Antigenicity, Allergenicity, Solubility, Toxicity and Physicochemical Properties of Vaccine construct 1 and Vaccine construct 2. (DOCX)

S6 Table Results of the secondary structure analysis of the vaccine constructs. (DOCX)

S7 Table Predicted conformational B cell epitopes residues of the designed multi-epitope based vaccines. (DOCX)

S8 Table Docking score of vaccine 1 and vaccine 2 against both TLR-3 and TLR-8. (DOCX)

## Abbreviations

DCC: Dynamic cross-correlation
DSSP: Definition of secondary structure of proteins
GRAVY: Grand average of hydropathicity
MD: Molecular Dynamics
PC: Principal component
Rg: Radius of gyration
RMSD: Root mean square deviation
RMSF: Root mean square fluctuation
TLR: Toll-like Receptor
