# Supplementary material for: In-silico formulation of a next-generation polyvalent vaccine against multiple strains of monkeypox virus and other related poxviruses
Source: PLoS One. 2024 May 17;19(5):e0300778. doi: 10.1371/journal.pone.0300778 (PMC11101047; doi:10.1371/journal.pone.0300778)
Supplement: S1 Fig — (DOCX) [file pone.0300778.s001.docx]

**
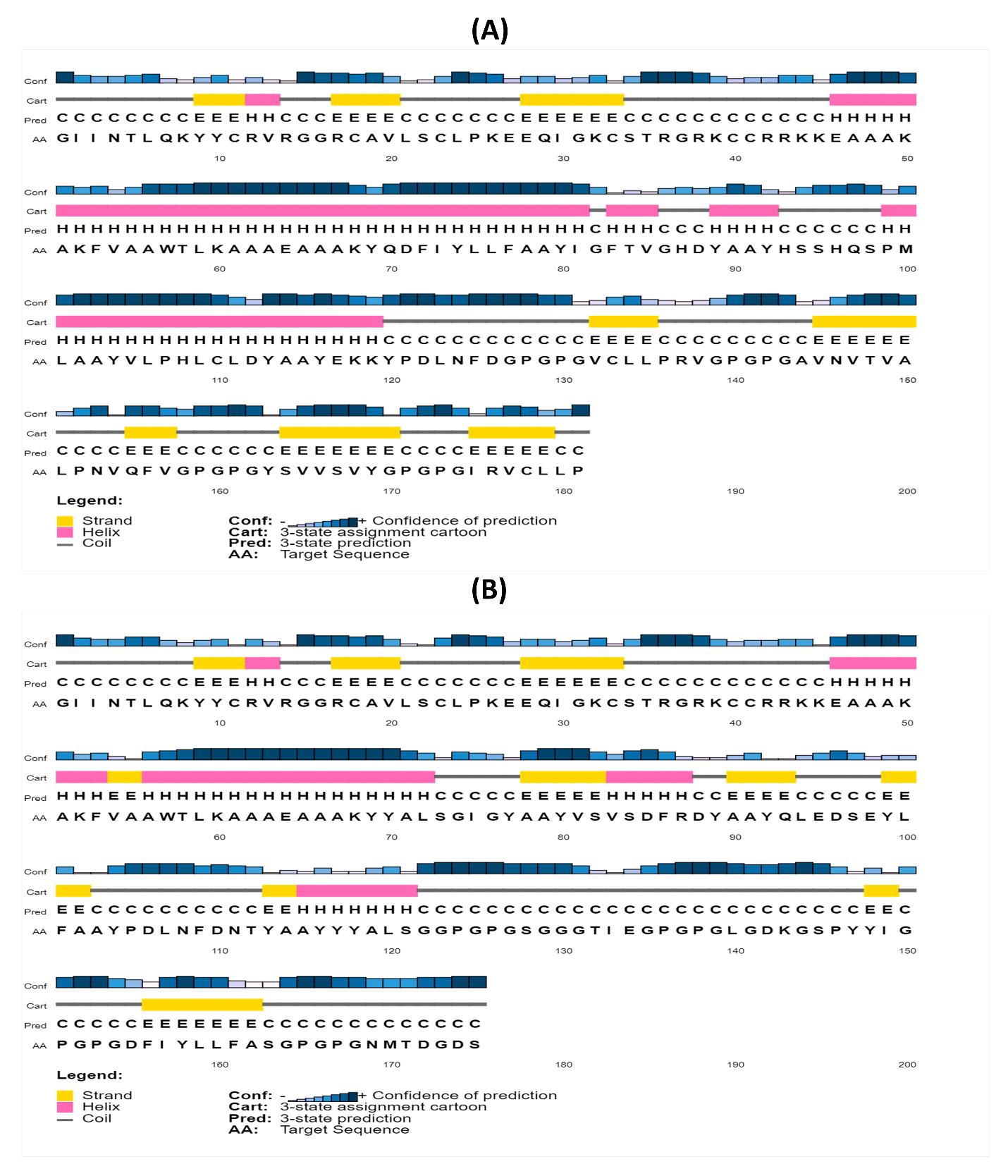
 S1 Figure:** Predicted secondary structures of (A) Vaccine Construct 1 and (B) Vaccine Construct 2 using PSIPRED.
