# Supplementary material for: In-silico formulation of a next-generation polyvalent vaccine against multiple strains of monkeypox virus and other related poxviruses
Source: PLoS One. 2024 May 17;19(5):e0300778. doi: 10.1371/journal.pone.0300778 (PMC11101047; doi:10.1371/journal.pone.0300778)
Supplement: S2 Fig — (DOCX) [file pone.0300778.s002.docx]

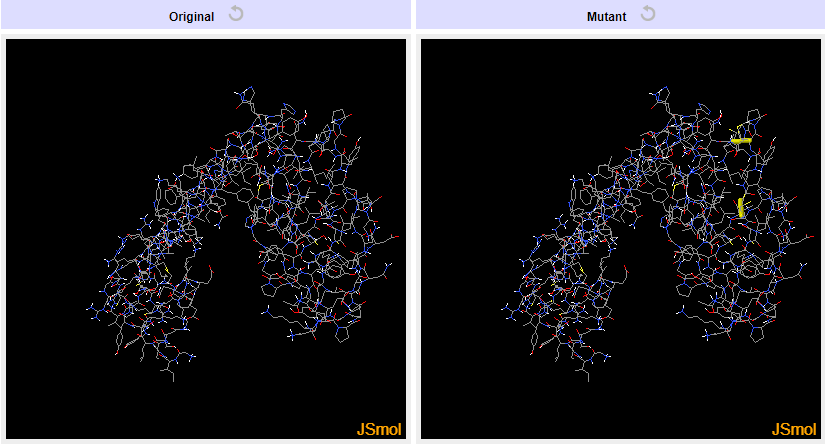


**B. Mutant Model**

**A. Original Model**

**S2 Figure**: Disulfide engineering study of the vaccine construct 1 (A) Original model (B) Mutated model.
