# Supplementary material for: In-silico formulation of a next-generation polyvalent vaccine against multiple strains of monkeypox virus and other related poxviruses
Source: PLoS One. 2024 May 17;19(5):e0300778. doi: 10.1371/journal.pone.0300778 (PMC11101047; doi:10.1371/journal.pone.0300778)
Supplement: S3 Fig — The red-colored portion represents the constructed vaccine. (DOCX) [file pone.0300778.s003.docx]

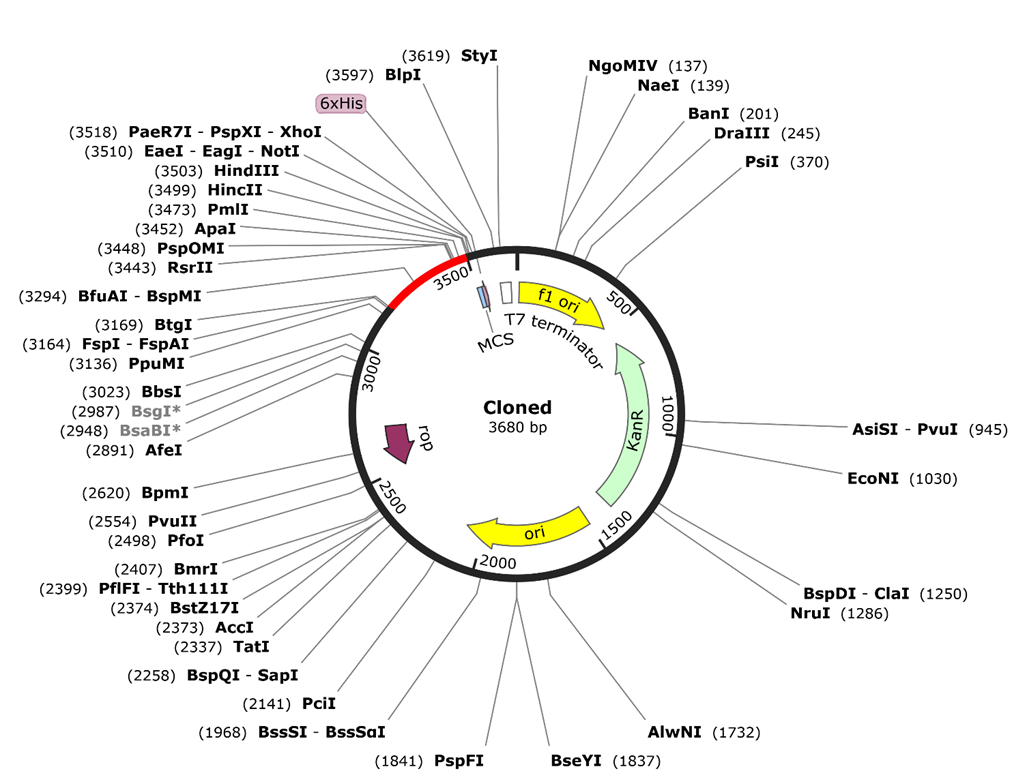


**S3 Figure:** The recombinant plasmid designed for mass production of the proposed vaccine. The red-colored portion represents the constructed vaccine.
