# Supplementary material for: In-silico formulation of a next-generation polyvalent vaccine against multiple strains of monkeypox virus and other related poxviruses
Source: PLoS One. 2024 May 17;19(5):e0300778. doi: 10.1371/journal.pone.0300778 (PMC11101047; doi:10.1371/journal.pone.0300778)
Supplement: S1 Table — (DOCX) [file pone.0300778.s004.docx]

**S1 Table:** Similar proteins among Monkeypox virus and Variola virus (smallpox)

| **Protein Sequence**  **(MPV)** | **BLASTp Percent Identity** | **Matched Protein Name**  **(Variola Virus)** | **Matched Protein Sequence ID**  **(Variola Virus)** | **E value** |
| --- | --- | --- | --- | --- |
| >YP_010377118.1 Virion core protein P4a [MPV] | 98.09% | p4a precursor of IMV core protein 4a  [Variola virus] | \|ABF23487.1\| | 0.0 |
| >USS79443.1 A5L [MPV] | 91.49% | TPA: 39kDa core protein  [Variola virus] | \|DAA80614.1\| | 1e^-178^ |
| >UTG40742.1 MPXVgp048 [MPV] | 95.67% | Hypothetical protein VARV_BEN68_59_043 [Variola virus] | \|ABF22803.1\| | 1e^-166^ |
| >UTG40861.1 MPXVgp165 [MPV] | 87.10% | Schlafen-like protein [Variola virus] | \|ABU91880.1\| | 2e^-53^ |
| >AIE40587.1 CD47-like putative membrane protein [MPV] | 95.31% | Hypothetical protein [Variola virus] | \|APR62885.1\| | 0.0 |
