# Supplementary material for: In-silico formulation of a next-generation polyvalent vaccine against multiple strains of monkeypox virus and other related poxviruses
Source: PLoS One. 2024 May 17;19(5):e0300778. doi: 10.1371/journal.pone.0300778 (PMC11101047; doi:10.1371/journal.pone.0300778)
Supplement: S2 Table — (DOCX) [file pone.0300778.s005.docx]

**S2 Table:** List of Potential T cell epitopes with their antigenic score, allergenecity, toxicity, transmembrane topology and conservancy analysis with their interacting MHC-1 alleles

| **Epitope** | **Antigenicity** | **Allergenicity** | **Conservancy** | **Toxicity** | **Transmembrane Topology** | **Prediction**  **score** | **Interacting MHC-I allele with an affinity of IC50 < 200nm** |
| --- | --- | --- | --- | --- | --- | --- | --- |
| IGFTVGHDY | 1.2537 | Non-Allergen | Conserved | Non-toxin | Outside | 0.5726 | HLA-C*12:03  HLA-C*03:03  HLA-A*30:02  HLA-A*29:02  HLA-C*14:02 |
| HSSHQSPML | 0.5356 | Non-Allergen | Conserved | Non-toxin | Outside | 0.6206 | HLA-C*03:03  HLA-C*15:02  HLA-C*12:03  HLA-B*15:02 |
| YYALSGIGY | 1.0229 | Non-Allergen | Conserved | Non-toxin | Outside | 1.6239 | HLA-C*14:02, HLA-B*15:02, HLA-C*03:03, HLA-A*29:02, HLA-C*12:03, HLA-C*07:02 |
| MDSMEALEY | 0.6215 | Non-Allergen | Conserved | Non-toxin | Outside | 1.0988 | HLA-C*12:03, HLA-C*03:03, HLA-B*35:01, HLA-A*29:02 |
| YQDFIYLLF | 0.6519 | Non-Allergen | Conserved | Non-toxin | Outside | 1.0543 | HLA-C*05:01, HLA-C*12:03, HLA-A*02:06, HLA-A*23:01 |
| VLPHLCLDY | 0.4183 | Non-Allergen | Conserved | Non-toxin | Outside | 0.5753 | HLA-C*12:03, HLA-C*14:02, HLA-A*29:02 |
| VSVSDFRDY | 1.2186 | Non-Allergen | Conserved | Non-toxin | Outside | 0.8878 | HLA-C*03:03, HLA-C*12:03, HLA-A*30:02 |
| QLEDSEYLF | 0.6677 | Non-Allergen | Conserved | Non-toxin | Outside | 0.6089 | HLA-C*05:01, HLA-B*15:02, HLA-C*12:03, HLA-C*03:03 |
| WDGIDYEEY | 0.9209 | Non-Allergen | Conserved | Non-toxin | Outside | 0.5121 | HLA-C*12:03 HLA-C*03:03 |
| PDLNFDNTY | 1.2952 | Non-Allergen | Conserved | Non-toxin | Outside | 0.5250 | HLA-C*12:03 HLA-C*03:03 |
| KLGDKGSPY | 1.7440 | Non-Allergen | Conserved | Non-toxin | Outside | 0.5461 | HLA-C*12:03  HLA-B*15:01  HLA-C*14:02  HLA-C*03:03  HLA-A*30:02 |
