# Supplementary material for: In-silico formulation of a next-generation polyvalent vaccine against multiple strains of monkeypox virus and other related poxviruses
Source: PLoS One. 2024 May 17;19(5):e0300778. doi: 10.1371/journal.pone.0300778 (PMC11101047; doi:10.1371/journal.pone.0300778)
Supplement: S3 Table — (DOCX) [file pone.0300778.s006.docx]

**S3 Table:** List of Potential B cell epitopes with their antigenic score, allergenecity, toxicity, transmembrane topology, conservancy analysis

| **Epitope** | **start** | **end** | **Antigenic score** | **Topology** | **Allergenicity** | **Toxicity** | **Conservancy** |
| --- | --- | --- | --- | --- | --- | --- | --- |
| EKKYPDLNFD | 161 | 170 | 1.5382 | Outside | Non-allergen | Non-toxin | Conserved |
| VCLLPRV | 426 | 432 | 1.1098 | Outside | Non-allergen | Non-toxin | Conserved |
| AVNVTVALPNVQFV | 327 | 340 | 1.0419 | Outside | Non-allergen | Non-toxin | Conserved |
| YSVVSVY | 31 | 37 | 1.0216 | Outside | Non-allergen | Non-toxin | Conserved |
| IRVCLLP | 424 | 430 | 1.0126 | Outside | Non-allergen | Non-toxin | Conserved |
| YYALSG | 97 | 102 | 0.9821 | Outside | Non-allergen | Non-toxin | Conserved |
| SGGGTIE | 218 | 224 | 0.8924 | Outside | Non-allergen | Non-toxin | Conserved |
| LGDKGSPYYI | 505 | 514 | 0.8794 | Outside | Non-allergen | Non-toxin | Conserved |
| DFIYLLFAS | 521 | 529 | 0.8396 | Outside | Non-allergen | Non-toxin | Conserved |
| NMTDGDS | 879 | 885 | 0.7020 | Outside | Non-allergen | Non-toxin | Conserved |
