# Supplementary material for: In-silico formulation of a next-generation polyvalent vaccine against multiple strains of monkeypox virus and other related poxviruses
Source: PLoS One. 2024 May 17;19(5):e0300778. doi: 10.1371/journal.pone.0300778 (PMC11101047; doi:10.1371/journal.pone.0300778)
Supplement: S4 Table — (DOCX) [file pone.0300778.s007.docx]

**S4 Table**: List of the epitopes selected for vaccine construction (selection criteria: Docking score, antigenicity, nonallergenicity, transmembrane topology, nontoxicity and conservancy)

| **Protein Name** | **T cell epitopes** | **B cell epitopes** |
| --- | --- | --- |
| MPXVgp165 | IGFTVGHDY | YYALSG |
|  | HSSHQSPML | SGGGTIE |
|  | YYALSGIGY | YSVVSVY |
| Virion core protein P4a | YQDFIYLLF | EKKYPDLNFD |
|  | VLPHLCLDY | VCLLPRV |
|  | VSVSDFRDY | AVNVTVALPNVQFV |
|  | QLEDSEYLF | IRVCLLP |
|  | PDLNFDNTY | LGDKGSPYYI |
|  |  | DFIYLLFAS |
|  |  | NMTDGDS |
