# Supplementary material for: In-silico formulation of a next-generation polyvalent vaccine against multiple strains of monkeypox virus and other related poxviruses
Source: PLoS One. 2024 May 17;19(5):e0300778. doi: 10.1371/journal.pone.0300778 (PMC11101047; doi:10.1371/journal.pone.0300778)
Supplement: S5 Table — (DOCX) [file pone.0300778.s008.docx]

**S5 Table:** Antigenicity, Allergenicity, Solubility, Toxicity and Physicochemical Properties of Vaccine construct 1 and Vaccine construct 2.

| **Features** | **Vaccine 1** | **Vaccine 2** |
| --- | --- | --- |
| Antigenicity (Threshold 0.4) | Antigenic (score: 0.5364) | Antigenic (score: 0.5458) |
| Allergenicity | Non- allergenic | Non- allergenic |
| Solubility | Soluble (score: 0.713) | Soluble (score: 0.567) |
| Toxicity | Non-toxic | Non-toxic |
| Number of amino acids | 181 | 175 |
| Theoretical Isoelectric point (pI) | 9.29 | 8.45 |
| Formula | C_885_H_1374_N_238_O_236_S_10_ | C_832_H_1254_N_218_O_249_S_7_ |
| Total number of atoms | 2743 | 2560 |
| (Asp + Glu) | 10 | 16 |
| (Arg + Lys) | 21 | 19 |
| Half-life | 30 hours (mammalian reticulocytes, in vitro).  >20 hours (yeast, in vivo).  >10 hours (Escherichia coli, in vivo). | 30 hours (mammalian reticulocytes, in vitro).  >20 hours (yeast, in vivo).  >10 hours (Escherichia coli, in vivo) |
| Aliphatic index | 85.75 | 64.34 |
| Instability index | 31.02 | 36.57 |
| GRAVY | 0.069 | -0.277 |
