# Supplementary material for: In-silico formulation of a next-generation polyvalent vaccine against multiple strains of monkeypox virus and other related poxviruses
Source: PLoS One. 2024 May 17;19(5):e0300778. doi: 10.1371/journal.pone.0300778 (PMC11101047; doi:10.1371/journal.pone.0300778)
Supplement: S6 Table — (DOCX) [file pone.0300778.s009.docx]

**S6 Table:** Results of the secondary structure analysis of the vaccine constructs.

| **Vaccine** | **Secondary Structure Element** | **SOPMA** |
| --- | --- | --- |
| **Vaccine 1** | Random Coil | 39.78% |
|  | Alpha Helix | 30.94% |
|  | Extended Strand | 25.41% |
|  | Beta Turn | 3.87% |
| **Vaccine 2** | Random Coil | 39.43% |
|  | Alpha Helix | 33.14% |
|  | Extended Strand | 20% |
|  | Beta Turn | 7.43% |
