# Supplementary material for: In-silico formulation of a next-generation polyvalent vaccine against multiple strains of monkeypox virus and other related poxviruses
Source: PLoS One. 2024 May 17;19(5):e0300778. doi: 10.1371/journal.pone.0300778 (PMC11101047; doi:10.1371/journal.pone.0300778)
Supplement: S7 Table — (DOCX) [file pone.0300778.s010.docx]

**S7 Table**: Predicted conformational B cell epitopes residues of the designed multi-epitope based vaccines

| **Vaccine** | **No.** | **Residues** | **Number of residues** | **Score** |
| --- | --- | --- | --- | --- |
| Vaccine construct 1 | 1 | A:G1, A:I2, A:I3, A:N4, A:T5, A:L6, A:Q7, A:K8, A:Y9, A:Y10, A:C11, A:V13, A:R14, A:G15, A:G16, A:R17, A:C18, A:A19, A:V20, A:S22, A:C23, A:L24, A:E27, A:Q29, A:I30, A:G31, A:K32, A:C33, A:S34, A:T35, A:R36, A:G37, A:R38, A:K39, A:C40, A:C41, A:R42, A:R43, A:K45, A:E46 | 40 | 0.74 |
|  | 2 | A:G86, A:H87, A:Y89, A:A90, A:A91, A:Y92, A:H93, A:S94, A:S95, A:H96, A:Q97, A:S98, A:P99, A:M100, A:L101, A:Y104 | 16 | 0.712 |
|  | 3 | A:L111, A:D112, A:Y113, A:A114, A:A115, A:E117, A:K118, A:K119, A:Y120, A:P121, A:D122, A:L123, A:N124, A:F125, A:D126, A:G127, A:C133, A:P136, A:R137, A:V138, A:G139, A:P140, A:G141, A:P142, A:G143, A:A144, A:V145, A:N146, A:V147, A:T148, A:V149, A:A150, A:L151, A:P152, A:N153, A:G170, A:P171, A:G172, A:P173, A:G174 | 40 | 0.63 |
| Vaccine construct 2 | 1 | A:G1, A:I2, A:I3, A:N4, A:T5, A:L6, A:Q7, A:K8, A:G15, A:G16, A:R17, A:C18, A:A19, A:V20, A:L21, A:S22, A:C23, A:L24, A:P25, A:K26, A:E27, A:Q29, A:G31, A:K32, A:C33, A:S34, A:T35, A:R36, A:G37, A:R38, A:K39, A:C41, A:R43 | 33 | 0.788 |
|  | 2 | A:A71, A:L72, A:S73, A:G74, A:I75, A:G76, A:Y77, A:A78, A:A79, A:Y80, A:S145, A:F161, A:G164, A:P165, A:G166, A:P167, A:G168, A:N169, A:M170, A:T171, A:D172, A:G173, A:D174, A:S175 | 24 | 0.715 |
|  | 3 | A:A102, A:A103, A:Y104 | 3 | 0.629 |
|  | 4 | A:P124, A:G125, A:P126, A:G127, A:S128, A:G129, A:G130, A:G131, A:T132, A:I133, A:E134, A:G135, A:P136, A:G137, A:I149, A:G150, A:P151, A:G152, A:P153 | 19 | 0.614 |
|  | 5 | A:V81, A:S82, A:V83, A:S84, A:D85, A:D88, A:G122, A:G139, A:L140, A:G141, A:D142, A:K143, A:G144 | 13 | 0.509 |
