# Supplementary material for: In-silico formulation of a next-generation polyvalent vaccine against multiple strains of monkeypox virus and other related poxviruses
Source: PLoS One. 2024 May 17;19(5):e0300778. doi: 10.1371/journal.pone.0300778 (PMC11101047; doi:10.1371/journal.pone.0300778)
Supplement: S8 Table — (DOCX) [file pone.0300778.s011.docx]

**S8 Table**: Docking score of vaccine 1 and vaccine 2 against both TLR-3 and TLR-8

| **TLR** | **Vaccine** | **H-Dock**  **Docking Score** | **ClusPro**  **Docking Energy** |
| --- | --- | --- | --- |
| TLR-3 | v-1 | -319.72 | -1061.8 |
|  | v-2 | -290.46 | -832.0 |
| TLR-8 | v-1 | -373.92 | -1176.6 |
|  | v-2 | -344.71 | -1055.7 |
